# Bis(pyridin-2-ylmeth­yl)ammonium nitrate

**DOI:** 10.1107/S1600536813008593

**Published:** 2013-04-05

**Authors:** Abubak’r Abrahams, Bernardus van Brecht, Richard Betz

**Affiliations:** a Nelson Mandela Metropolitan University, Summerstrand Campus, Department of Chemistry, University Way, Summerstrand, PO Box 77000, Port Elizabeth, 6031, South Africa

## Abstract

In the title compound, C_12_H_14_N_3_^+^·NO_3_^−^, the mononitrate of protonated bis­(pyridin-2-ylmeth­yl)amine, the least-squares planes defined by the non-H atoms of the two aromatic moieties inter­sect at an angle of 7.91 (6)°. In the crystal, N—H⋯N, N—H⋯O and C—H⋯N hydrogen bonds, as well as C—H⋯O contacts, connect the entities into a three-dimensional network. The shortest centroid–centroid distance between two aromatic systems is 3.7255 (8) Å and is apparent between the two different aromatic moieties.

## Related literature

For the crystal structure of the trinitrate of bis­(pyridin-2-ylmeth­yl)amine, see: Junk *et al.* (2006 ▶). For graph-set analysis of hydrogen bonds, see: Etter *et al.* (1990 ▶); Bernstein *et al.* (1995 ▶).
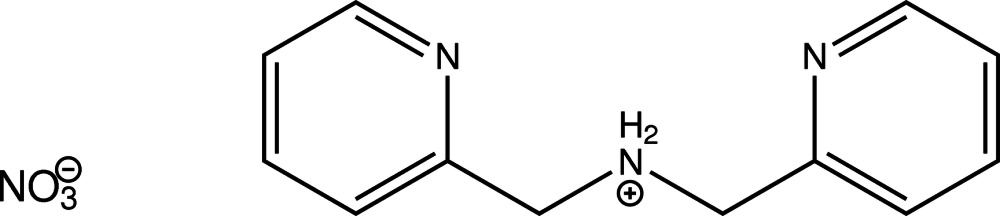


## Experimental

### 

#### Crystal data

C_12_H_14_N_3_^+^·NO_3_^−^
*M* *_r_* = 262.27
Orthorhombic, 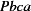

*a* = 11.2236 (2) Å
*b* = 13.4714 (4) Å
*c* = 16.5303 (4) Å
*V* = 2499.34 (11) Å^3^
*Z* = 8
Mo *K*α radiation
μ = 0.10 mm^−1^
*T* = 200 K
0.50 × 0.35 × 0.24 mm

#### Data collection

Bruker APEXII CCD diffractometer
Absorption correction: multi-scan (*SADABS*; Bruker, 2001 ▶) *T*_min_ = 0.923, *T*_max_ = 1.000
12816 measured reflections
3082 independent reflections
2568 reflections with *I* > 2σ(*I*)
*R*_int_ = 0.014

#### Refinement

*R*[*F* ^2^ > 2σ(*F* ^2^)] = 0.041
*wR*(*F* ^2^) = 0.122
*S* = 1.04
3082 reflections
180 parameters
H atoms treated by a mixture of independent and constrained refinement
Δρ_max_ = 0.29 e Å^−3^
Δρ_min_ = −0.26 e Å^−3^

### 

Data collection: *APEX2* (Bruker, 2007 ▶); cell refinement: *SAINT* (Bruker, 2007 ▶); data reduction: *SAINT*; program(s) used to solve structure: *SHELXS97* (Sheldrick, 2008 ▶); program(s) used to refine structure: *SHELXL97* (Sheldrick, 2008 ▶); molecular graphics: *ORTEP-3 for Windows* (Farrugia, 2012 ▶) and *Mercury* (Macrae *et al.*, 2008 ▶); software used to prepare material for publication: *SHELXL97* and *PLATON* (Spek, 2009 ▶).

## Supplementary Material

Crystal structure: contains datablock(s) I, global. DOI: 10.1107/S1600536813008593/vn2068sup1.cif

Structure factors: contains datablock(s) I. DOI: 10.1107/S1600536813008593/vn2068Isup2.hkl

Supplementary material file. DOI: 10.1107/S1600536813008593/vn2068Isup3.cdx

Supplementary material file. DOI: 10.1107/S1600536813008593/vn2068Isup4.cml

Additional supplementary materials:  crystallographic information; 3D view; checkCIF report

## Figures and Tables

**Table 1:** Hydrogen-bond geometry (Å, °)

*D*—H⋯*A*	*D*—H	H⋯*A*	*D*⋯*A*	*D*—H⋯*A*
C13—H13⋯N2	0.95	2.61	3.5339 (18)	164
N1—H71⋯O3^i^	0.893 (18)	2.349 (18)	3.0721 (15)	138.2 (14)
N1—H72⋯O2^ii^	0.902 (19)	1.982 (19)	2.8831 (15)	176.1 (15)
N1—H72⋯O1^ii^	0.902 (19)	2.584 (18)	3.2201 (16)	128.1 (14)
N1—H72⋯N2^ii^	0.902 (19)	2.651 (18)	3.4905 (15)	155.1 (14)
C15—H15⋯O2^iii^	0.95	2.39	3.1858 (19)	141
C1—H1*A*⋯O1^iv^	0.99	2.54	3.5132 (18)	167
C25—H25⋯O1^v^	0.95	2.53	3.3959 (16)	151
C24—H24⋯O2^vi^	0.95	2.55	3.4620 (16)	161

Symmetry codes: (i) 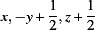; (ii) 
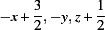
; (iii) 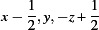; (iv) 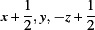; (v) 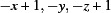; (vi) 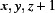.

**Fig. 1.**
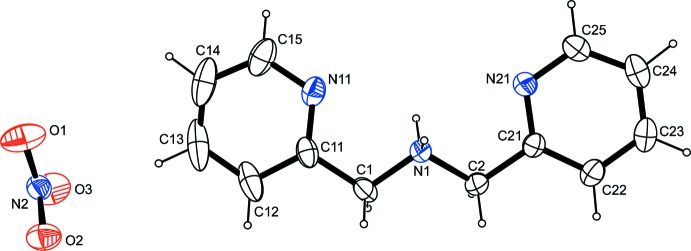
The molecular structure of the title compound, with atom labels and anisotropic displacement ellipsoids (drawn at 50% probability level).

**Fig. 2.**
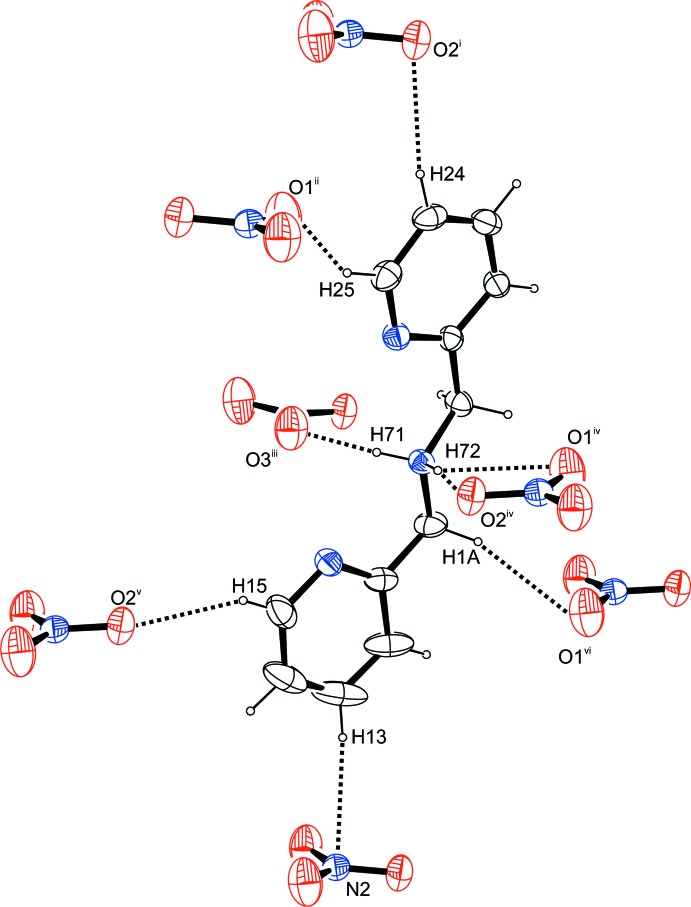
Intermolecular contacts, viewed approximately along [1 1 0]. Symmetry operators: ix, y, z + 1; ii -x + 1, -y, -z + 1; iiix, -y + 1/2, z + 1/2; iv -x + 3/2, -y, z + 1/2; vx - 1/2, y, -z + 1/2; vix + 1/2, y, -z + 1/2.

**Fig. 3.**
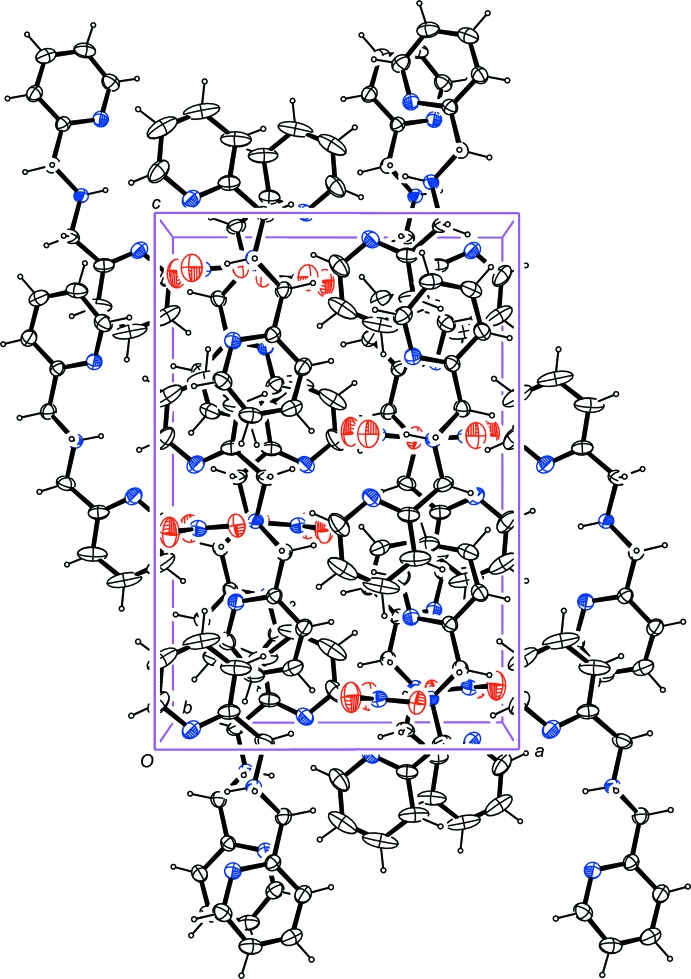
Molecular packing of the title compound, viewed along [0 1 0] (anisotropic displacement ellipsoids drawn at 50% probability level).

## supplementary crystallographic information

### Comment

Upon the attempted synthesis of a rare-earth metal coordination compound applying bis(pyridin-2-ylmethyl)amine as an auxilliary ligand, the title compound was unintentionally obtained as the only crystalline reaction product. The crystal structure of the trinitrate salt of bis(pyridin-2-ylmethyl)amine has been reported earlier (Junk *et al.*, 2006).

The twofold-protonated amine-type nitrogen atom is present in a tetrahedral coordination environment. The angles set up by the atoms connected to it cover a range of 107.7 (11)–111.81 (9) °. The least-squares planes defined by the respective non-hydrogen atoms of the two aromatic moieties enclose an angle of 7.91 (6) ° (Fig. 1).

In the crystal, classical hydrogen bonds of the N–H···O type are observed next to C–H···O contacts whose range falls by more than 0.1 Å below the sum of van-der-Waals radii of the atoms participating. In addition, a C–H···N contact involving the nitrate anion is apparent. One of the N–H···O hydrogen bonds shows bifurcation. All the aforementioned contacts are exclusively established between atoms on the cation as well as the anion. Metrical parameters as well as information about the symmetry of these contacts are summarized in Table 1. In total, the entities of the crystal structure are connected to a three-dimensional network. In terms of graph-set analysis (Etter *et al.*, 1990; Bernstein *et al.*, 1995), the descriptor for the C–H···O contacts is *DDDD* on the unary level while the classical hydrogen bonds necessitate a *DDD* descriptor on the same level if the bifurcated hydrogen bond is counted as two separate hydrogen bonds. The shortest intercentroid distance between two aromatic systems is measured at 3.7255 (8) Å and is apparent between the two different aromatic moieties (Fig. 2).

The packing of the title compound in the crystal structure is shown in Figure 3.

### Experimental

Lanthanum nitrate was reacted with bis(pyridin-2-ylmethyl)amine in water. Upon free evaporation of the solvent, a crystalline solid was obtained from which irregular-shaped white crystals could be selected.

### Refinement

Carbon-bound H atoms were placed in calculated positions (C–H 0.95 Å for aromatic carbon atoms and C–H 0.99 Å for methylene groups) and were included in the refinement in the riding model approximation, with *U*(H) set to 1.2*U*_eq_(C). Both nitrogen-bound H atoms were located on a difference Fourier map and refined freely.

### Crystal data

**Table:**

C_12_H_14_N_3_^+^·NO_3_^−^	*F*(000) = 1104
*M**_r_* = 262.27	*D*_x_ = 1.394 Mg m^−^^3^
Orthorhombic, *P**b**c**a*	Mo *K*α radiation, λ = 0.71073 Å
Hall symbol: -P 2ac 2ab	Cell parameters from 5628 reflections
*a* = 11.2236 (2) Å	θ = 2.5–28.2°
*b* = 13.4714 (4) Å	µ = 0.10 mm^−^^1^
*c* = 16.5303 (4) Å	*T* = 200 K
*V* = 2499.34 (11) Å^3^	Irregular, white
*Z* = 8	0.50 × 0.35 × 0.24 mm

### Data collection

**Table:**

Bruker APEXII CCD diffractometer	3082 independent reflections
Radiation source: fine-focus sealed tube	2568 reflections with *I* > 2σ(*I*)
Graphite monochromator	*R*_int_ = 0.014
φ and ω scans	θ_max_ = 28.3°, θ_min_ = 2.7°
Absorption correction: multi-scan (*SADABS*; Bruker, 2001)	*h* = −8→14
*T*_min_ = 0.923, *T*_max_ = 1.000	*k* = −17→17
12816 measured reflections	*l* = −22→17

### Refinement

**Table:**

Refinement on *F*^2^	Primary atom site location: structure-invariant direct methods
Least-squares matrix: full	Secondary atom site location: difference Fourier map
*R*[*F*^2^ > 2σ(*F*^2^)] = 0.041	Hydrogen site location: inferred from neighbouring sites
*wR*(*F*^2^) = 0.122	H atoms treated by a mixture of independent and constrained refinement
*S* = 1.04	*w* = 1/[σ^2^(*F*_o_^2^) + (0.0695*P*)^2^ + 0.627*P*] where *P* = (*F*_o_^2^ + 2*F*_c_^2^)/3
3082 reflections	(Δ/σ)_max_ < 0.001
180 parameters	Δρ_max_ = 0.29 e Å^−^^3^
0 restraints	Δρ_min_ = −0.26 e Å^−^^3^

### Fractional atomic coordinates and isotropic or equivalent isotropic displacement parameters (Å^2^)

**Table:**

	*x*	*y*	*z*	*U*_iso_*/*U*_eq_	
O1	0.53822 (10)	0.03915 (9)	0.09542 (8)	0.0595 (3)	
O2	0.72195 (8)	0.07826 (8)	0.08342 (6)	0.0438 (3)	
O3	0.58787 (10)	0.19315 (8)	0.09035 (8)	0.0540 (3)	
N1	0.76754 (9)	0.13548 (8)	0.57810 (6)	0.0257 (2)	
H71	0.6936 (16)	0.1564 (13)	0.5883 (10)	0.044 (4)*	
H72	0.7725 (15)	0.0688 (14)	0.5820 (10)	0.044 (4)*	
N2	0.61491 (9)	0.10403 (8)	0.08911 (6)	0.0300 (2)	
N11	0.59551 (10)	0.11038 (8)	0.46858 (6)	0.0345 (2)	
N21	0.71063 (9)	0.11508 (7)	0.73540 (6)	0.0297 (2)	
C1	0.79740 (12)	0.16440 (10)	0.49410 (7)	0.0351 (3)	
H1A	0.8717	0.1305	0.4774	0.042*	
H1B	0.8116	0.2369	0.4919	0.042*	
C2	0.84972 (10)	0.18126 (9)	0.63756 (7)	0.0295 (2)	
H2A	0.8442	0.2544	0.6335	0.035*	
H2B	0.9326	0.1617	0.6246	0.035*	
C11	0.69911 (12)	0.13770 (8)	0.43630 (7)	0.0313 (3)	
C12	0.71806 (18)	0.14422 (10)	0.35348 (8)	0.0507 (4)	
H12	0.7937	0.1623	0.3324	0.061*	
C13	0.6227 (2)	0.12345 (11)	0.30224 (9)	0.0657 (6)	
H13	0.6315	0.1291	0.2453	0.079*	
C14	0.51552 (19)	0.09468 (12)	0.33522 (11)	0.0598 (5)	
H14	0.4496	0.0794	0.3014	0.072*	
C15	0.50553 (14)	0.08849 (11)	0.41766 (10)	0.0472 (4)	
H15	0.4316	0.0678	0.4400	0.057*	
C21	0.82056 (10)	0.14965 (8)	0.72268 (7)	0.0255 (2)	
C22	0.90516 (11)	0.16031 (9)	0.78369 (7)	0.0309 (3)	
H22	0.9828	0.1843	0.7719	0.037*	
C23	0.87333 (12)	0.13503 (9)	0.86208 (7)	0.0359 (3)	
H23	0.9287	0.1417	0.9052	0.043*	
C24	0.75986 (13)	0.10001 (9)	0.87633 (7)	0.0366 (3)	
H24	0.7353	0.0829	0.9295	0.044*	
C25	0.68233 (11)	0.09025 (9)	0.81153 (7)	0.0346 (3)	
H25	0.6049	0.0646	0.8216	0.041*	

### Atomic displacement parameters (Å^2^)

**Table:**

	*U*^11^	*U*^22^	*U*^33^	*U*^12^	*U*^13^	*U*^23^
O1	0.0397 (6)	0.0467 (6)	0.0920 (9)	−0.0119 (5)	−0.0001 (5)	0.0126 (6)
O2	0.0298 (5)	0.0473 (6)	0.0542 (6)	0.0045 (4)	0.0046 (4)	−0.0013 (4)
O3	0.0484 (6)	0.0330 (5)	0.0805 (8)	0.0060 (4)	−0.0001 (5)	0.0027 (5)
N1	0.0254 (5)	0.0290 (5)	0.0229 (4)	−0.0029 (4)	−0.0008 (3)	0.0030 (3)
N2	0.0309 (5)	0.0333 (5)	0.0260 (5)	0.0003 (4)	0.0013 (4)	0.0017 (4)
N11	0.0339 (5)	0.0374 (5)	0.0322 (5)	0.0069 (4)	−0.0073 (4)	−0.0051 (4)
N21	0.0281 (5)	0.0325 (5)	0.0284 (5)	−0.0005 (4)	0.0000 (4)	0.0034 (4)
C1	0.0395 (6)	0.0421 (6)	0.0236 (5)	−0.0092 (5)	0.0062 (5)	0.0033 (5)
C2	0.0270 (5)	0.0327 (6)	0.0287 (6)	−0.0068 (4)	−0.0022 (4)	0.0014 (4)
C11	0.0494 (7)	0.0219 (5)	0.0225 (5)	0.0018 (5)	−0.0017 (5)	0.0013 (4)
C12	0.0989 (13)	0.0300 (6)	0.0233 (6)	−0.0127 (7)	0.0039 (7)	0.0010 (5)
C13	0.138 (2)	0.0328 (7)	0.0259 (7)	0.0027 (9)	−0.0235 (9)	−0.0003 (5)
C14	0.0905 (14)	0.0376 (7)	0.0513 (9)	0.0206 (8)	−0.0415 (9)	−0.0113 (7)
C15	0.0444 (8)	0.0432 (7)	0.0542 (9)	0.0160 (6)	−0.0214 (6)	−0.0127 (6)
C21	0.0259 (5)	0.0244 (5)	0.0263 (5)	0.0028 (4)	−0.0025 (4)	−0.0014 (4)
C22	0.0289 (5)	0.0323 (6)	0.0316 (6)	0.0044 (4)	−0.0048 (4)	−0.0051 (4)
C23	0.0464 (7)	0.0334 (6)	0.0278 (6)	0.0122 (5)	−0.0096 (5)	−0.0052 (5)
C24	0.0546 (8)	0.0303 (6)	0.0248 (5)	0.0119 (5)	0.0031 (5)	0.0020 (4)
C25	0.0367 (7)	0.0339 (6)	0.0332 (6)	0.0018 (5)	0.0063 (5)	0.0047 (5)

### Geometric parameters (Å, º)

**Table:**

O1—N2	1.2311 (15)	C11—C12	1.3883 (17)
O2—N2	1.2541 (14)	C12—C13	1.393 (3)
O3—N2	1.2385 (14)	C12—H12	0.9500
N1—C1	1.4806 (14)	C13—C14	1.376 (3)
N1—C2	1.4822 (14)	C13—H13	0.9500
N1—H71	0.893 (18)	C14—C15	1.370 (2)
N1—H72	0.902 (19)	C14—H14	0.9500
N11—C11	1.3313 (17)	C15—H15	0.9500
N11—C15	1.3473 (17)	C21—C22	1.3926 (15)
N21—C21	1.3354 (15)	C22—C23	1.3866 (18)
N21—C25	1.3403 (15)	C22—H22	0.9500
C1—C11	1.5030 (18)	C23—C24	1.378 (2)
C1—H1A	0.9900	C23—H23	0.9500
C1—H1B	0.9900	C24—C25	1.3864 (19)
C2—C21	1.5061 (16)	C24—H24	0.9500
C2—H2A	0.9900	C25—H25	0.9500
C2—H2B	0.9900		
			
C1—N1—C2	111.81 (9)	C11—C12—H12	121.0
C1—N1—H71	107.7 (11)	C13—C12—H12	121.0
C2—N1—H71	108.8 (11)	C14—C13—C12	119.15 (15)
C1—N1—H72	108.4 (10)	C14—C13—H13	120.4
C2—N1—H72	109.1 (10)	C12—C13—H13	120.4
H71—N1—H72	111.0 (15)	C15—C14—C13	118.86 (15)
O1—N2—O3	121.03 (11)	C15—C14—H14	120.6
O1—N2—O2	118.66 (11)	C13—C14—H14	120.6
O3—N2—O2	120.29 (11)	N11—C15—C14	123.15 (17)
C11—N11—C15	117.70 (13)	N11—C15—H15	118.4
C21—N21—C25	116.99 (10)	C14—C15—H15	118.4
N1—C1—C11	111.53 (10)	N21—C21—C22	123.50 (11)
N1—C1—H1A	109.3	N21—C21—C2	116.51 (10)
C11—C1—H1A	109.3	C22—C21—C2	119.95 (10)
N1—C1—H1B	109.3	C23—C22—C21	118.41 (12)
C11—C1—H1B	109.3	C23—C22—H22	120.8
H1A—C1—H1B	108.0	C21—C22—H22	120.8
N1—C2—C21	111.51 (9)	C24—C23—C22	118.81 (11)
N1—C2—H2A	109.3	C24—C23—H23	120.6
C21—C2—H2A	109.3	C22—C23—H23	120.6
N1—C2—H2B	109.3	C23—C24—C25	118.70 (11)
C21—C2—H2B	109.3	C23—C24—H24	120.6
H2A—C2—H2B	108.0	C25—C24—H24	120.6
N11—C11—C12	123.13 (13)	N21—C25—C24	123.58 (12)
N11—C11—C1	116.90 (10)	N21—C25—H25	118.2
C12—C11—C1	119.95 (13)	C24—C25—H25	118.2
C11—C12—C13	117.98 (17)		
			
C2—N1—C1—C11	−166.97 (10)	C13—C14—C15—N11	0.8 (2)
C1—N1—C2—C21	−178.69 (10)	C25—N21—C21—C22	0.28 (17)
C15—N11—C11—C12	−0.18 (18)	C25—N21—C21—C2	−177.56 (10)
C15—N11—C11—C1	178.44 (11)	N1—C2—C21—N21	−20.60 (14)
N1—C1—C11—N11	11.67 (16)	N1—C2—C21—C22	161.48 (10)
N1—C1—C11—C12	−169.68 (11)	N21—C21—C22—C23	−0.91 (17)
N11—C11—C12—C13	1.7 (2)	C2—C21—C22—C23	176.87 (10)
C1—C11—C12—C13	−176.91 (12)	C21—C22—C23—C24	0.33 (17)
C11—C12—C13—C14	−1.9 (2)	C22—C23—C24—C25	0.79 (17)
C12—C13—C14—C15	0.8 (2)	C21—N21—C25—C24	0.94 (18)
C11—N11—C15—C14	−1.1 (2)	C23—C24—C25—N21	−1.49 (19)

### Hydrogen-bond geometry (Å, º)

**Table:**

*D*—H···*A*	*D*—H	H···*A*	*D*···*A*	*D*—H···*A*
C13—H13···N2	0.95	2.61	3.5339 (18)	164
N1—H71···O3^i^	0.893 (18)	2.349 (18)	3.0721 (15)	138.2 (14)
N1—H72···O2^ii^	0.902 (19)	1.982 (19)	2.8831 (15)	176.1 (15)
N1—H72···O1^ii^	0.902 (19)	2.584 (18)	3.2201 (16)	128.1 (14)
N1—H72···N2^ii^	0.902 (19)	2.651 (18)	3.4905 (15)	155.1 (14)
C15—H15···O2^iii^	0.95	2.39	3.1858 (19)	141
C1—H1*A*···O1^iv^	0.99	2.54	3.5132 (18)	167
C25—H25···O1^v^	0.95	2.53	3.3959 (16)	151
C24—H24···O2^vi^	0.95	2.55	3.4620 (16)	161

Symmetry codes: (i) *x*, −*y*+1/2, *z*+1/2; (ii) −*x*+3/2, −*y*, *z*+1/2; (iii) *x*−1/2, *y*, −*z*+1/2; (iv) *x*+1/2, *y*, −*z*+1/2; (v) −*x*+1, −*y*, −*z*+1; (vi) *x*, *y*, *z*+1.
